# Physical Enhancement? Nanocarrier? Current Progress in Transdermal Drug Delivery

**DOI:** 10.3390/nano11020335

**Published:** 2021-01-28

**Authors:** Noriyuki Uchida, Masayoshi Yanagi, Hiroki Hamada

**Affiliations:** 1 Department of Applied Chemistry, Graduate School of Engineering, Tokyo University of Agriculture and Technology, 2-24-16 Nakacho, Koganei, Tokyo 184-8588, Japan; 2 RIKEN Center for Emergent Matter Science, 2-1 Hirosawa, Wako, Saitama 351-0198, Japan; 3 Department of Life Science, Faculty of Science, Okayama University of Science, 1-1 Ridai Kita, Okayama 700-0005, Japan; s14l087ym@ous.jp

**Keywords:** transdermal drug delivery, skin, physical enhancement, microneedle, nanocarrier

## Abstract

A transdermal drug delivery system (TDDS) is a method that provides drug adsorption via the skin. TDDS could replace conventional oral administration and blood administration because it is easily accessible. However, it is still difficult to design efficient TDDS due to the high barrier property of skin covered with stratum corneum, which inhibits the permeation of drug molecules. Thus far, TDDS methods by applying physical stimuli such as microneedles and chemical stimuli such as surfactants have been actively developed. However, it has been hard to avoid inflammation at the administration site because these methods partially destroy the skin tissue. On the other hand, TDDS with nanocarriers minimizing damage to the skin tissues has emerged together with the development of nanotechnology in recent years. This review focuses on current trends in TDDS.

## 1. Introduction

**Figure 1:**
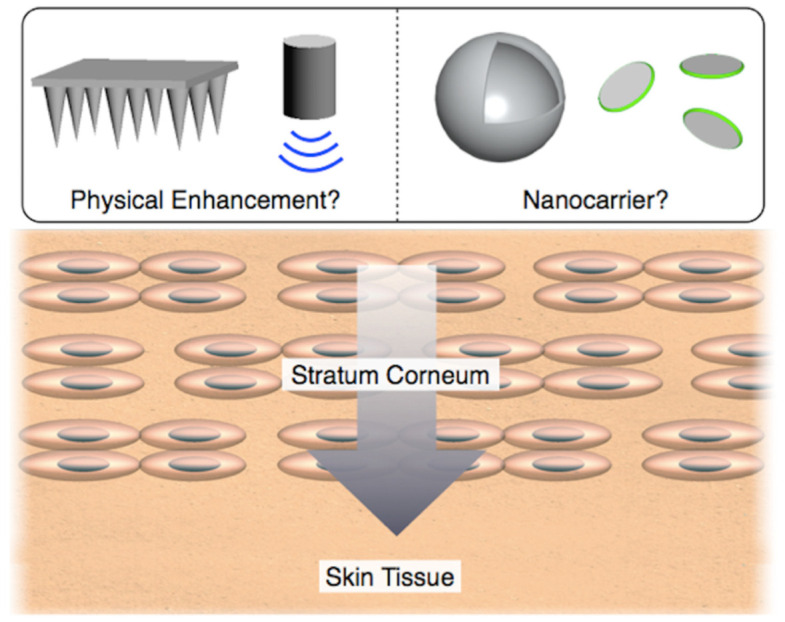
Schematic illustration of representative strategies in transdermal drug delivery system (TDDS) using physical enhancements and nanocarriers.

A lot of effort has been paid to achieve efficient drug delivery into skin tissue for decades. The transdermal drug delivery system (TDDS) is an attractive administration method because it is easily accessible [1,2,3,4,5]. However, a major part of drugs is still administered with needles due to the skin barrier. In general, needle injections have two serious problems. One is pain and needle phobia, and the other is infections due to the reuse of needles and unintended damage. Injections are unpleasant and painful, increasing needle phobia and reducing quality of life. In fact, some studies suggested that 30% of adults suffer from needle phobia [6,7,8,9]. A more significant risk is unintended needle injury for both the patient and the doctors. Hundreds of millions of dollars have been provided for additional health care, and transmission of infectious diseases has increased [10,11]. In particular, 800,000 needle injuries by medical professionals were reported in the United States each year [11,12]. Nevertheless, needle injections are still mainstream for overcoming the skin barriers. Remarkable barrier properties of the skin tissue are mainly due to the stratum corneum (SC), which represents the thin outer layer of the epidermis [13,14,15]. Differently from other tissues in the body, the stratum corneum is composed of corneocytes that are surrounded by an extracellular environment of lipids assembled as multiple lamellar bilayers. These lipids prevent excessive water loss from the body and block the entry of hydrophobic drugs with low molecular weight. This offers a significant challenge to drug administration via the skin tissue as systemic therapy following their entry into superficial dermal capillaries. As a result, various methods of skin permeation to enhance the transport of drugs through the SC have been investigated (Figure 1). As a first-generation of TDDS, the delivery of small lipophilic drugs has been reported, and the number of clinical applications is still increasing. After that, the development of second-generation TDDS using ultrasound [16,17,18,19,20], iontophoresis [21,22,23,24,25,26,27], and chemical enhancers [28,29], has emerged, which produced clinical products. Especially, iontophoresis can control the supply rate in real-time, realizing a wide range of applications. In addition, a third-generation TDDS by using electroporation [30,31] and microneedles [32,33,34,35,36,37,38,39,40,41,42,43,44,45,46,47,48,49,50,51], has been developed for minimizing the effect on the stratum corneum in the skin barrier. In particular, microneedles have been actively investigated, and clinical trials of delivery of macromolecules such as insulin, parathyroid hormone, and influenza vaccines were currently performed. Recently, nanocarrier-based TDDS [52,53,54,55,56,57,58,59,60,61,62,63,64,65,66,67,68,69,70,71,72,73,74,75,76,77,78,79,80,81,82,83,84,85,86,87,88,89,90,91,92,93,94,95,96,97,98,99,100,101,102] have attracted particular attention, aiming to minimize the unfavorable effects on the skin [103,104,105,106]. It is revealed that drug molecules could be delivered to the skin tissue by controlling the flexibility, shape, and size of the nanocarriers, suggesting the potentials for fourth-generation TDDS. This review introduces recent trends in TDDS with the developments of such nanomaterials.

## 2. Transdermal Drug Delivery by Physical Enhancement

### 2.1. Sonophoresis

Sonophoresis is a transdermal delivery method in which the absorption of drugs occurs into the epidermis, dermis, and skin appendages using ultrasound. Hydrophilic molecules and macromolecules are used as drugs. Sonophoresis occurs when ultrasound waves stimulate micro-vibrations within the skin epidermis increasing the kinetic energy of molecules. This technology is effective at low frequencies and it is widely utilized in hospitals for transdermal delivery. As a pioneer work, Langer et al. succeeded in transdermal delivery of proteins by using sonophoresis [18]. Recently, Boddu et al. reported that sonophoresis could be used for the delivery of econazole nitrate for treating Raynaud’s phenomenon [19]. In addition, Shende et al. reported that the combination of sonophoresis and transdermal patches could be used to treat rheumatoid arthritis [20].

### 2.2. Iontophoresis

**Figure 2:**
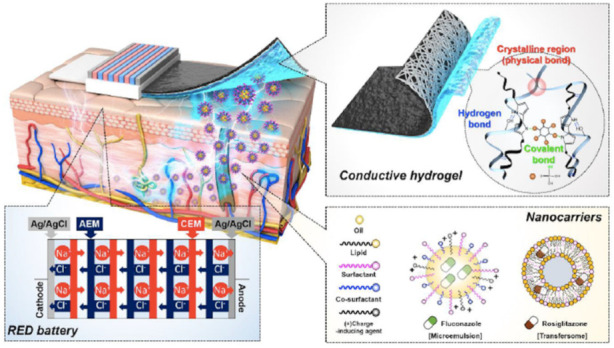
Schematic of the reverse electrodialysis (RED)-driven iontophoretic patch and each component in which the RED battery is operated by the transport of Na^+^ and Cl^−^ ions. The polypyrrole-appended polyvinyl alcohol electroconductive hydrogel patches are constructed with the RED system. Reprinted with permission from ref 24. Copyright 2020 American Chemical Society.

Iontophoresis is a transdermal delivery method that uses an electric current to deliver a substance to the skin tissue to break the limitations of the skin barrier, which usually allows for penetration of only fat-soluble substances with a molecular weight of about 500. The most important requirement for iontophoresis is that the drug molecules should be ionized to be water-soluble. Iontophoresis allows for the delivery of molecules that are unsuitable for passive delivery across the stratum corneum. Although the requirements for the formulation are clearly different from those of lipophilic molecules delivered by the conventional transdermal delivery method, uncharged and water-insoluble molecules can also be used for iontophoresis by appropriate modulation of their physicochemical properties [21]. Penbutolol is a non-cardioselective agent for adrenoreceptor blocking, which is used for the treatment of hypertension. Tablet is the conventional dosage form for penbutolol, which has limitations such as high incidence of adverse effects because of variable absorption profile and hepatic first-pass metabolism. To address these problems, Ita et al. succeeded in TDDS of penbutolol sulfate by iontophoresis [22]. Ranitidine is used in pediatric medicine, especially in intensive care, and is prescribed in a variety of clinical indications such as gastro-esophageal reflux disease, benign gastric and duodenal ulcerations, for which gastric acid reduction is needed. Oral and intravenous delivery have been used as an administration method. Especially in children, the oral bioavailability of ranitidine is highly variable. Moreover, some drugs contain alcohol, and no oral preparation is licensed for use in children under 3 years of age. For overcoming these limitations, Charro et al. reported transdermal delivery of ranitidine by iontophoresis [23]. More recently, Hwang et al. successfully developed an iontophoretic method coupled with hydrogel embedding nanocarriers (Figure 2) [24]. Reverse electrodialysis (RED)-driven iontophoretic patch was constructed with polypyrrole-appended polyvinyl alcohol electroconductive hydrogel. Electrically mobile drug nanocarriers can efficiently be delivered by this method. Thus far, iontophoretic therapeutic systems such as lidocaine (LidoSite^®^; 2004), fentanyl (Ionsys^®^; 2006), and sumatriptan (Zecuity^®^; 2013) have been approved.

### 2.3. Chemical Penetration Enhancer

TDDS using chemical penetration enhancers is one of the most widely used approaches. In 1997, Buyuktimkin et al. evaluated water, hydrocarbons, alkanols, acids, esters, alkylamino esters, ureas, amides, sulfoxides, terpenes, steroids, dioxolanes, pyrrolidones, and imidazole derivatives as penetration enhancers for human skin [28,29]. Shah et al. suggested that the enhancers work by the following mechanisms; (1) increase of diffusivity of the drug in the skin, (2) fluidization of SC, which causes a decrease in barrier function, (3) increasing the thermodynamic activity of the drug in the carrier, and (4) affecting the partition coefficient of the drug. Although about 150 kinds of chemical penetration enhancers have been used in the pharmaceutical industry thus far, they are not ideal in terms of toxicity, irritation, allergy, and sensitization, and further improvement is desired.

### 2.4. Electroporation

In contrast to iontophoresis, high voltage pulses of 50–500 V is applied in electroporation to perturb lipid organization in the stratum corneum. Electrochemotherapy is the most successful clinical application of electroporation, which is used for the treatment of cutaneous tumors in malignant melanoma patients [33]. In addition, Mohand et al. recently reported that effective transdermal delivery can be achieved by electroporation of non-ionic surfactant-based vesicles containing *Annona squamosa* [34]. Because the carrier is non-ionized, electroporation enhanced skin permeability with little effect on the organization of the vesicle.

### 2.5. Microneedle

**Figure 3:**
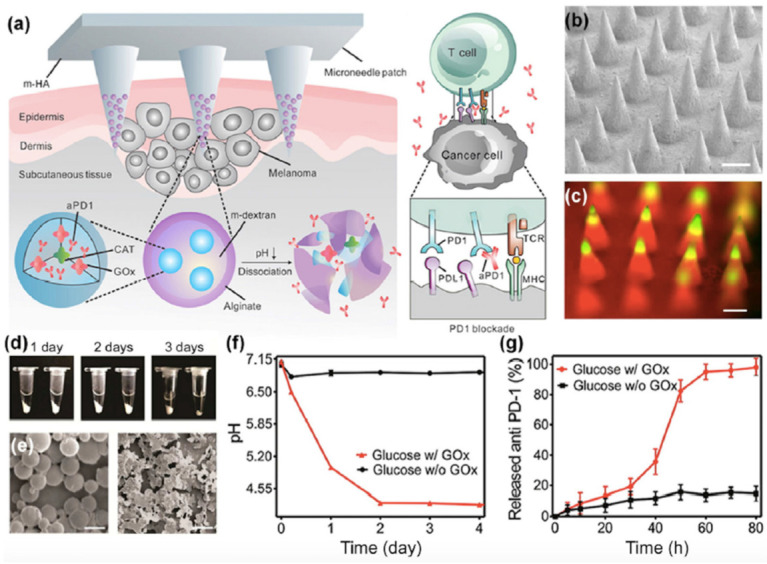
(**a**) Schematic design of the TDDS of the anti-PD1 antibody by the microneedle patch. (**b**) SEM image and (**c**) fluorescence image of the microneedles. (**d**) Appearance and (**e**) SEM images of the anti-PD1 antibody-loaded nanoparticles. (**f**,**g**) Release profiles of the anti-PD1 antibody from the microneedle in the presence of glucose oxidase. Reprinted with permission from ref 54. Copyright 2016 American Chemical Society.

Microneedles are micron-scale devices, which enable mechanical perforation of the skin barrier. Because the microneedle can penetrate the stratum corneum and epidermis without reaching the pain-sensitive nerve cells [47]. This technology can be mainly divided into four categories as follows; (i) solid microneedles that are used to pretreat the skin prior to formulation application, (ii) drug-coated microneedles with drugs on the microneedle surface for rapid transdermal delivery method to the epidermis, (iii) dissolving microneedles in which drug molecules are formulated into the microneedle, and (iv) hollow microneedles, which are in contact with a drug reservoir. Microneedles are one of the most extensively studied transdermal delivery method in the category of physical perturbation. For example, Oomens et al. recently reported the calculation of diffusion and kinetics for vaccine delivery to figure out the optimal geometry of microneedles using a computer model [48]. In addition, Prausnitz et al. found that transdermal delivery for the eye can be designed by applying an optimal surface coating to microneedles [50]. Transdermal delivery of vaccines is one of the attractive applications because microneedles also enable the delivery of biomacromolecules. Gu et al. reported delivery of anti-PD1 antibodies by using a microneedle for cancer immunotherapy (Figure 3) [54]. In this work, self-degradable microneedles composed of biocompatible hyaluronic acid matrix integrated with acid-degradable dextran nanoparticles encapsulating programmed death-1 protein (aPD1) were used.

From a historical point of view, physical enhancement methods have been improved with a focus on how to minimize damage to the skin. In particular, microneedle methods are currently capable of transdermal delivery with low invasiveness, and related research would be further expanded in the future.

## 3. Transdermal Drug Delivery Using Nanocarrier

Not only the above-mentioned TDDS using physical enhancements, TDDS with nanocarriers has emerged together with the recent progress in nanotechnology for minimizing damage to the skin tissues.

### 3.1. Flexible Liposome

**Figure 4:**
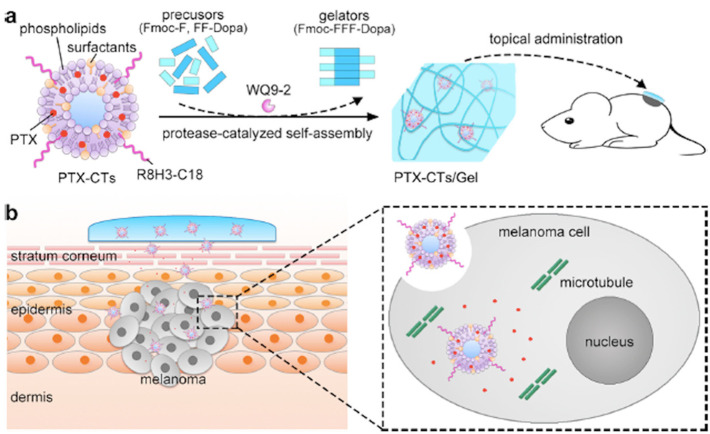
(**a**) Schematic illustration of the preparation and the application of a liposome embedding hydrogel as a paintable patch for TDDS. (**b**) Schematic illustration of the enhancement on the efficiency of TDDS for noninvasive chemotherapy of melanoma. Reprinted with permission from ref 63. Copyright 2018 American Chemical Society.

The flexibility of liposomes affects the efficiency of TDDS [55,56,57,58,59,60,61,62,63,64,65]. In 1992, Cevc et al. reported highly deformable liposomes, so-called transfersomes, which penetrate intact skin [55]. After that, flexible liposomes containing ethanol showing high skin permeation capability have emerged. Atopic eczema is one of the inflammatory skin diseases, and it represents serious problems for patients and physicians as well as for researchers. The characteristic point of this disease is an itchy red rash that favors the skin creases such as the folds of elbows. Atopic eczema is characterized by an increase in activity of mast cells, higher expression levels of IgE antibodies, and a decrease in the production of ceramides at the molecular level. Nicotinamide (NIC) is an important coenzyme and involved in various oxidation-reduction reactions in biological systems as an antioxidant by inhibiting poly-adenosine diphosphate-ribose polymerase. The effects are derived from inhibition of poly-adenosine diphosphate-ribose polymerase, and NIC increasingly gains interest in the treatment of several skin diseases. Sayed et al. proposed a new approach of TDDS of NIC using an ethosome for the treatment of atopic eczema [56]. Ibuprofen is a popular drug for the treatment of fever and pain. Ibuprofen is widely used in both adults and children thanks to its high effectiveness and low-side-effect. It belongs to the NSAID family and shows pharmacodynamic actions by inhibiting COX-1 and COX-2, which are the enzymes related to inflammatory pathways and the formation of pro-inflammatory prostaglandins. TDDS of ibuprofen could avoid side effects associated with gastrointestinal ulceration and bleeding due to the local effect on the production of mucosal prostaglandins. In addition, TDDS could benefit pediatric patients often suffering from taking the full dose of oral medication and vomiting [62]. More recently, Mo et al. successfully developed a peptide hydrogel embedding ethanol-containing liposomes for chemotherapy of melanoma (Figure 4) [63].

### 3.2. Lipid Nanoparticle

Lipid nanoparticles have been used as an alternative TDDS for pharmaceutical drugs and cosmetic compounds since the early 1990s [66,67,68,69,70,71,72,73,74,75,76,77,78,79,80,81,82]. A solid lipid nanoparticle (SLN) was prepared by the exchange of the liquid lipid oil of the emulsions by solid lipid, which remains at a solid-state at room temperature and body temperature [66,67]. The SLNs are useful for incorporating lipophilic, hydrophilic, and water-insoluble compounds owing to their structural characteristics. Nanostructured lipid carriers (NLC) are known as the second generation of lipid nanoparticles [68,69,70,71,72,73,74,75]. A variety of amphiphilic molecules can be used to prepare NLC ranging from phospholipids to artificial surfactants. Different from SLN, the lipid matrix of NLC is composed of a blend of solid and liquid lipids, which reduce the melting point of the solid lipid by keeping the solid matrix at room and body temperatures. NLC can also be stabilized in aqueous dispersion using a surfactant. For the improvement of these lipid-based nanocarriers, Park et al. developed lipid nanoparticles composed of ceramide and succeeded in TDDS of apigenin [76]. Gratieri et al. reported clobetasol-loaded lipid nanocarriers and investigated the effects on hair follicles [77]. In addition, Hamada et al. successfully prepared ultra-small nanoparticles by using anionic phospholipids. It was revealed that ultra-small lipid nanoparticles encapsulating antioxidant resveratrol [80] and anticancer paclitaxel [81] exhibited high skin permeation capability.

### 3.3. Bicelle

**Figure 5:**
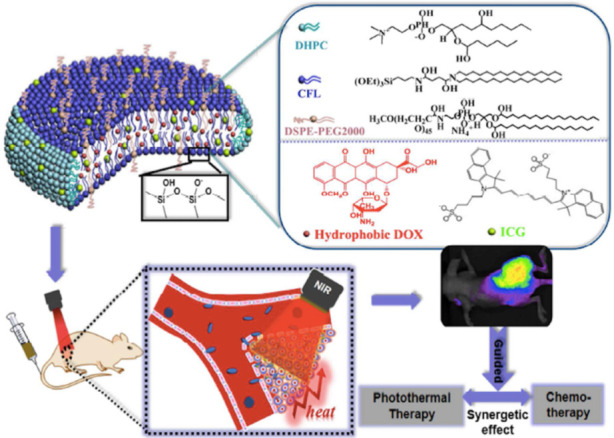
Schematic illustration of the bicelle containing doxorubicin (DOX) and fluorescent dye. Reprinted with permission from ref 88. Copyright 2017 American Chemical Society.

The Bicelle is a disk-shaped phospholipid assembly and it has been actively studied in recent years in several research fields due to its unique shape [83,84,85,86,87,88,89,90,91]. Bicelles are formed by mixing long-chain and short-chain phospholipids in an aqueous solution. As a typical example, a bicelle structure is formed with a diameter of about 15 to 50 nm by stabilizing the edge of dipalmitoylphosphatidylcholine (DMPC) bilayers with short-chain dihexanoylphosphatidylcholine (DHPC) [83]. Firstly, bicelle has attracted attention as alignment media in NMR measurements, taking advantage of the magnetic field orientation due to its anisotropic shape. For example, Ramamoorthy et al. systematically investigated the effects of chain length to form the bicelles for the use in NMR analysis of membrane proteins [83]. Recently, applications of the bicelles to TDDS have emerged. The first reason for this is that disk-shaped carriers were found to show stronger adhesion to microvascular networks [107] and longer circulation times [108] than spherical carriers. In addition, Lopez et al. reported that the unique two-dimensional shape of bicelles is advantageous because the carriers have to pass through narrow gaps (6–10 nm) between skin cells to penetrate deep inside skin tissue [85]. Vesicles normally have diameters of more than 100 nm and are too large to pass through the narrow gaps. In the case of small micelles, the carriers tend to undergo cellular uptake, which prevents penetration into skin tissues. On the other hand, bicelles are sufficiently thin (<4 nm) to pass through intercellular gaps but are wide enough (20–1000 nm) to resist cellular uptake. However, the bicelle is generally an unstable self-assembly and tends to transform in skin tissue when applied to TDDS, and various studies have been reported to solve this problem [86]. Dai et al. succeeded in the preparation of kinetically stable bicelles by covalent fixation of bilayers with polymerizable phospholipids. By encapsulating an anticancer drug, doxorubicin (DOX), in this bicelle and administering it to mice, DOX was successfully delivered in vivo (Figure 5) [88]. In addition, Ishida et al. realized a kinetically stable and temperature-responsive bicelle using a kinetically stable gel-phase phospholipid bilayer and chemically designed surfactants and applied them to the delivery of DOX [90].

### 3.4. Nanoemulsion

Nanoemulsions are dispersions of water and oil phases stabilized by surfactants with a diameter in the submicron range [92,93,94,95,96,97,98]. Nanoemulsions can be prepared by high-energy and low-energy emulsification techniques and usually contain a relatively low amount of surfactant. The low surfactant content brings advantages in topical application with low irritation to the skin. The small-sized droplet provides a large surface area and uniform distribution on the skin, resulting in good occlusiveness, aesthetic qualities, film formation, and skin feel [92,93]. For the effective use of emulsion, Harwansh et al. reported efficient TDDS of the herbal drug by using a nanoemulsion [94]. In addition, Casiraghi et al. evaluated the effects of the size of emulsion on TDDS and revealed that nanometer-sized emulsion carriers efficiently delivered apolar molecules into skin tissue [97].

### 3.5. Other Nanocarriers

In addition to the above-mentioned examples, nanocarriers for TDDS, which exhibit unique functions, have been developed [99,100,101,102,103,104]. Dendrimer is a dendritic macromolecule, and it can be used for TDDS. In particular, lower generation cationic dendrimers are more effective in enhancing skin permeation by interacting with the skin lipid bilayers. Polyamidoamine (PAMAM) dendrimers are shown to deliver drugs such as 5-fluorouracil, riboflavin, indomethacin, and ketoprofen [100]. Nanogels are nanocarriers consisting of a network of hydrophilic polymers that are chemically or physically crosslinked and swell in a preferred medium. Lyer et al. created pH-responsive nanogels and demonstrated in vivo and ex vivo studies that they are effective for the treatment of skin cancers [101]. Cyclodextrin-based TDDS have also been developed [109,110]. For example, Kilimozhi et al. succeeded in the co-delivery of curcumin and resveratrol using a cyclodextrin nanosponge [109].

As mentioned above, various nanocarriers have been developed because it is easy to prepare rather than physical enhancement methods. The key for the clinical applications of the nanocarriers is how to design materials that pass through the skin barrier. The effects of flexibility, shape, and size of nanocarriers have been evaluated, and ideal properties have been clarified. In the future, the development of nanocarriers that combine these factors would be expected.

## 4. Conclusions

In this paper, we have introduced recent trends in TDDS by classifying them into methods that use physical perturbations and nanocarriers. TDDS by using physical perturbations has already been put into practical use, and preparations are being made for the social implementation of TDDS. If a promising material for TDDS would be developed by using nanocarriers, it could be an innovative treatment for the next generation of skin diseases.
